# Maternal immunoglobulins are distributed in the offspring’s brain to support the maintenance of cortical interneurons in the postnatal period

**DOI:** 10.1186/s41232-024-00336-3

**Published:** 2024-05-15

**Authors:** Keiko Morimoto, Rikuo Takahashi, Goro Takahashi, Michio Miyajima, Kazunori Nakajima

**Affiliations:** Department of Anatomy, Keio University School of Medicine, 35 Shinanomachi, Shinjuku-Ku, Tokyo, 160-8582 Japan

**Keywords:** Immunoglobulin, Microglia, Interneuron

## Abstract

It is known that maternal immunoglobulins (Igs) are transferred to the offspring across the placenta. However, receiving maternal Igs, especially before the blood–brain barrier (BBB) is formed in the offspring’s brain, carries the risk of transferring some brain-reactive Igs. It is thus hypothesized that there may be some unknown benefit to the offspring’s brain that overweighs this risk. In this study, we show that the Ig detected in the embryonic/perinatal mouse brain is IgG not produced by the pups themselves, but is basically transferred from the mother across the placenta using the neonatal Fc receptor (FcRn) during embryonic stages. The amount of IgG in the brain gradually decreases after birth, and almost disappears within 3 weeks postnatally. IgG is detected on axon bundles, microglia, and some meningeal cells, including border-associated macrophages (BAMs), endothelial cells, and fibroblasts. Using *Fcer1g* knock-out (KO) mice, we show that BAMs and microglia receive maternal IgG in an Fc receptor γ chain (FcRγ)-dependent manner, but IgG on other meningeal cells and axon bundles is received independently of the FcRγ. These results suggest that maternal IgG may be used in multiple ways by different mechanisms. In maternal IgG-deficient mice, the number of interneurons in the cerebral cortex is not altered around birth but is reduced postnatally, suggesting that receipt of maternal IgG is necessary for the maintenance of cortical interneurons in the postnatal period. These data suggest that maternal IgG has an important function in the developing brain, where neither obvious inflammation nor infection is observed.

**Supplementary Information:**

The online version contains supplementary material available at 10.1186/s41232-024-00336-3.

## Background

Embryos are highly sensitive to various stimuli and are protected by maternal molecules that cross the placenta. One such molecule thought to be important in preventing infection is immunoglobulin (Ig). Immunoglobulin G (IgG) is the only Ig that can be transferred from the mother to the fetus using the neonatal Fc receptor (FcRn, encoded by *Fcgrt*) from the late first trimester in humans, exceeding maternal IgG levels at term [1].

During normal pregnancy, embryos are considered almost sterile, although several studies have detected microbial presence in human placenta and fetal samples [2]. On the contrary, receiving maternal IgG carries risks; epidemiologic evidence suggests that autoimmune diseases are associated with an increased risk of neurodevelopmental disorders such as autism spectrum disorder (ASD), attention-deficit/hyperactivity disorder (ADHD), Tourette syndrome in the offspring [3]. In addition, maternal brain reactive Ig is present in 10–20% of mothers of a child with ASD and Igs against contactin-associated protein 2 (CASPR2), collapsin response mediator protein 1 (CRMP1), stress-induced phosphoprotein 1 (STIP1), lactate dehydrogenase A, lactate dehydrogenase B, Y-box binding protein 1 (YBX1), and guanine deaminase are known as ASD-related antibodies [4].

During development, the blood–brain barrier (BBB) is immature, allowing many plasma proteins, including Igs, to freely enter the brain parenchyma until E17.5 in mice [5]. Interestingly, the period when the brain contains a significant amount of IgG coincides with crucial developmental events, including the generation and migration of neurons and glial cells, angiogenesis, synaptogenesis, pruning, and myelination [6]. The absence of infection and inflammation in the healthy developing brain raises questions about the role of IgG beyond immunological protection. In this study, we investigated the localization of brain IgG and its time course during development. We found that IgG in the developing brain is derived from maternal sources. We show that the IgG in the developing brain is detected in axon bundles, microglia, and meningeal cells and that the receptors involved in IgG capture are either dependent or independent of Fc receptor γ chain (FcRγ). In maternal IgG-deficient mice, we observe a reduction in the number of cortical interneurons at postnatal stages, suggesting a previously unrecognized pivotal role of maternal IgG in the developing brain beyond its conventional immunoprotective function.

## Methods

### Experimental animals

Pregnant ICR and C57BL/6 J mice were purchased from Japan SLC (Hamamatsu, Japan). B6.Cg-*Rag2*^*tm1.1Cgn*^*/*J (Strain number: 008449) and B6.129P(Cg)-*Cx3cr1*^*tm1Litt*^/J (Strain number: 005582) mice were obtained from The Jackson Laboratory (Bar Harbor, ME). *Aldh1l1*-GFP mice [7] (MMRRC, Stock number: 011015) were obtained from the University of California, Davis. GAD67-GFP knock-in (KI) ICR mice were kindly provided by Dr. Yuchio Yanagawa [8]. The day of vaginal plug detection was considered embryonic day (E) 0. Animal care and experiments were performed under the control of the Keio University Institutional Animal Care and Use Committee in accordance with the Institutional Guidelines on Animal Experimentation at Keio University, the Japanese Government Law Concerning the Protection and Control of Animals, and the Japanese Government Notification of Feeding and Safekeeping of Animals.

### Generation of *Fcgrt*^*− /−*^ and *Fcer1g*^*− /−*^ mice

*Fcgrt*^*−/−*^ and *Fcer1g*^*−/−*^ mice were generated using the improved genome editing via the oviductal nucleic acid delivery (i-GONAD) method [9] (Additional file 1: Figure S1A, 1B). CRISPR guide RNAs were designed using the IDT website (https://sg.idtdna.com/pages/products/crispr-genome-editing). i-GONAD was performed as previously described with minor modifications. Briefly, a mixture of 15 μM crRNA for Target 1 (see below), 15 μM crRNA for Target 2 (see below), 2 μg/μl ssODN (see below), 30 μM TracrRNA, 0.02% Fast Green and 1 μg/μl Cas 9 Nuclease (all from Integrated DNA Technologies, USA) was prepared in Opti-MEM at E0.7 and injected into the oviduct from upstream of the ampulla using a glass micropipette. Electroporation was performed using NEPA21 (NEPA GENE, Japan) (poring pulse 50 V, 5 ms pulse interval, 3 pulses, 10% decay, single pulse orientation, and transfer pulse 10 V, 50 ms pulse, 50 ms pulse interval, 3 pulses, 40% decay, ± pulse orientation). Mice with expected deletion of the target gene were screened by electrophoresis of the PCR products and further confirmed by sequencing. Genotyping primers are listed below.

### For *Fcgrt*^−^^/−^ mouse

crRNA target1: CTGGTCTACGAAGAGTC

crRNA target2: GGGCCAAATTTATGTGG

ssODN: CTGTCTGTCGTCTTGGACTGGGTCTCCATCCCACCATCCAGCGTCCTGGTCTACGAAGAGTCGAATTCCCACATAAATTTGGCCCCAAATCTGTGTGTGCATCGTTATTCTCAAGTTTCAAGCAGCTGGA

genotyping primers:

*Fcgrt* genotyping common Fwd: AGGTGAAAGTTCACAGAGGAACACTC

*Fcgrt* genotyping wild type Rev: CACTCTTTGGCTCTTCTTCCTGTCC

*Fcgrt* genotyping KO Rev: GGCCAAATTTATGTGGGACTCTTCG

Expected size of the PCR products: wild type 297 bp, KO 104 bp.

### For* Fcer1g*^*−/−*^mouse

crRNA target1: GTGTCTAGCGGAGTCTCTAG

crRNA target2: GTAAGTCTTTAACGGAGATG

ssODN: TGGGTATATAAAGTTCTAGATAGGAAGGTAAGGGTTATGGTGGGTGTCTAGCGGAGTCTCGAATTCCTCCGTTAAAGACTTACTCACTGACATTTCTCTTCTTCCAGCCTCCTTTGCTTCATTTCT

genotyping primers:

*Fcer1g* genotyping common Fwd: TCTTGGTGTCTTGCTGCCTTT

*Fcer1g* genotyping wild type Rev: GCCTTCCCCTTACCTGCTTG

*Fcer1g* genotyping KO Rev: TTAACGGAGGAATTCGAGACTCC

Expected size of the PCR products: wild type 653 bp, KO 261 bp.

### Immunostaining

Mice were perfused with ice-cold 4% paraformaldehyde (PFA) in 0.1 M sodium phosphate buffer (pH 7.4), and their brains were further fixed with 4% PFA at 4 ℃ with gentle agitation for 2 h. The brains were cryoprotected by sequential immersion in 20% and 30% sucrose in phosphate-buffered saline (PBS) (-) at 4 ℃ overnight, embedded in 75% O.C.T. compound (Sakura, Japan), and frozen with liquid nitrogen. Brains were cryosectioned coronally with a cryostat (CM3050 S; Leica, Germany) at 20 μm thick on MAS-coated glass slides (MAS-02: Matsunami Glass Ind., Ltd.). Samples were blocked with 10% normal goat or donkey serum in PBS containing 0.05% Triton X-100 (PBST) for 1 h at room temperature (RT) and incubated with primary antibodies at 4 ℃ overnight. After three washes with PBST, samples were incubated with species-specific secondary antibodies and 4,6-diamidino-2-phenylindole, dihydrochloride (DAPI; Invitrogen) (1:2000) for 2 h at RT. Fluorescent images were captured with a confocal laser scanning microscope (TCS SP8; Leica, Germany). Images were processed using Photoshop (Adobe Systems, San Jose, CA). The primary antibodies used were anti-Iba1 antibody (1:1000, 019–19741, WAKO), anti-P2RY12 (purinergic receptor P2Y, G-protein coupled 12) antibody (1:1000, AS-55043A, Anaspec), anti-mannose receptor antibody (1:600, ab64693, abcam), anti-CD31 antibody (1:1000, ab119341, abcam), anti-PDGFRβ antibody (1:100, MA5-15143, Invitrogen), anti-mouse FcγRIII antibody (1:200, AF1960, R&D systems), PE anti-mouse CD64 (FcγRI) antibody (1:100, 164403, BioLegend), anti-somatostatin (SST) antibody (H-106) (1:500, sc-13099, abcam), anti-parvalbumin (PV) antibody (1:1000, P3088, SIGMA), and anti-reelin antibody (1:400, AF3820, R&D systems). The secondary antibodies used were goat anti-mouse IgG (H + L) cross-adsorbed secondary antibody, Alexa Fluor™ 488 (1:1000, A11001, Invitrogen), goat anti-rabbit IgG (H + L) cross-adsorbed secondary antibody, Alexa Fluor™ 488 (1:1000, A11008, Invitrogen), goat anti-mouse IgG (H + L) cross-adsorbed secondary antibody, Alexa Fluor™ 594 (1:1000, A11005, Invitrogen), goat anti-rabbit IgG (H + L) cross-adsorbed secondary antibody, Alexa Fluor™ 594 (1:1000, A11012, Invitrogen), donkey anti-mouse IgG (H + L) highly cross-adsorbed secondary antibody, Alexa Fluor™ 488 (1:1000, A21202, Invitrogen), donkey anti-rabbit IgG (H + L) highly cross-adsorbed secondary antibody, Alexa Fluor™ 488 (1:1000, A32790, Invitrogen), donkey anti-goat IgG (H + L) cross-adsorbed secondary antibody, Alexa Fluor™ 647 (1:1000, A21447, Invitrogen), and AffiniPure goat anti-Armenian hamster IgG (H + L), Alexa Fluor™ 594 (1:1000, 127–585-160, Jackson).

Preabsorption experiments were performed by preincubating 2 μg/ml Alexa Fluor™ 594-labeled goat anti-mouse IgG (H + L) cross-adsorbed secondary antibody (A11005, Invitrogen) with 2 μg/ml purified mouse IgG (0107–01,SouthernBiotech) for 10 min at 4 ℃.

### Depletion of the endogenous IgG signal for immunohistochemistry

Four percent PFA-fixed cryosections were immersed in 10 mM sodium citrate (pH 6.0) containing 0.05% Tween 20 and autoclaved at 105 °C for 10 min.

### Perfusion of rabbit Igs

Pups were subjected to transcardiac perfusion with 0.5 mg/ml rabbit IgG (I-5006, Sigma) in Dulbecco’s PBS (-) (16220015, KOHJIN BIO). After 5 min, brains were removed and immersion fixed in 4% PFA in 0.1 M sodium phosphate buffer (pH 7.4) at 4 ℃ with gentle agitation for 2 h.

### Western blotting

Mice were perfused with ice-cold Dulbecco’s PBS (-) and the brain was dissected and lysed in lysis buffer (10 mM Tris–HCl [pH 7.4], 150 mM NaCl, 1% [vol/vol] Nonidet P40 Substitute, 0.5 mM EDTA) plus EDTA-free protease inhibitor cocktail cOmplete (Roche) and phosphatase inhibitor phosSTOP (Roche). Samples were separated by sodium dodecyl sulfate–polyacrylamide gel electrophoresis (SDS-PAGE) (Mini-Protean TGX Precastgels 4–20%, Bio-Rad, Richmond, CA), and transferred to polyvinyldifluoride (PVDF) membranes using the Trans-Blot Turbo Transfer system (Bio-Rad, Richmond, CA, USA). The membranes were blocked in 5% skim milk in PBST (0.05% Tween 20 in PBS) for 1 h and probed with the primary antibodies overnight and secondary antibodies for 1 h at RT. The following antibodies were used for Western blotting: Peroxidase AffiniPure goat anti-mouse IgG (H + L) antibody (1:10,000, 115–035-146, Jackson) and anti-GAPDH antibody (1:1000, sc32233, Santa Cruz Biotechnology). Bands were detected using Amersham ECL Prime Western Blotting Detection Reagents (GE Healthcare) and images were captured using LAS-4000 mini.

### Single-cell data analysis

A publicly available single-cell RNA-seq data was used to examine the expression of Fcγ receptors. The dataset was obtained from the whole brains of E7-E18.5 mouse embryos, and the annotated dataset was downloaded from the authors’ website (http://mousebrain.org/development/downloads.html) [10]. Single-cell data were processed and visualized using the Seurat R package (version 4) [11]. The original dataset was converted to a Seurat object from loom format using the as.Seurat function in SeuratDisk package (https://github.com/mojaveazure/seurat-disk), and the expression patterns were visualized using the DotPlot function and the VlnPlot function in the Seurat package.

### Cell count

Iba1 + cells in the cerebral cortex of *Fcgrt* heterozygous mice and *Fcgrt* KO mice at E14.5 and PV + , SST + , and reelin + cells in wild-type or *Fcgrt* KO mice and *Rag2* KO mice were counted manually. P2RY12-positive cells in *Fcgrt* heterozygous and *Fcgrt* KO mice at postnatal day (P) 0 were calculated by using Fiji [12] and CLIJ2 [13] plugins. After applying median and Gaussian filters for noise reduction, Z-stack images were binarized using the Otsu threshold. Connected component labeling was performed on the binarized images to identify cells, and abnormal cells were removed based on the voxel size of the labels. Finally, labeled cells within a manually defined ROI were counted to calculate cell density.

Bin analysis was performed by dividing the cortical wall between the meningeal surface and the ventricular surface into 10 equal regions (10 bins). The number of GFP + cells in each bin was calculated by using Fiji. In each image, the Despeckle and Watershed functions were applied to the GFP and DAPI channels and binarized with the same manually defined thresholds for all images. DAPI + regions within GFP + regions were automatically counted as GFP + cells, and the cell density within each bin was calculated.

### Statistical analysis

Quantitative data are presented as mean ± standard deviation (SD). Individual values are shown as circles in bar graphs. Statistical analyses were performed using Welch’s *t* test. Differences with *P* values < 0.05 were considered significant. *, *P* < 0.05; and **, *P* < 0.01.

## Results

### IgG in the developing mouse brain is of maternal origin and not produced in the embryos

**Fig. 1:**
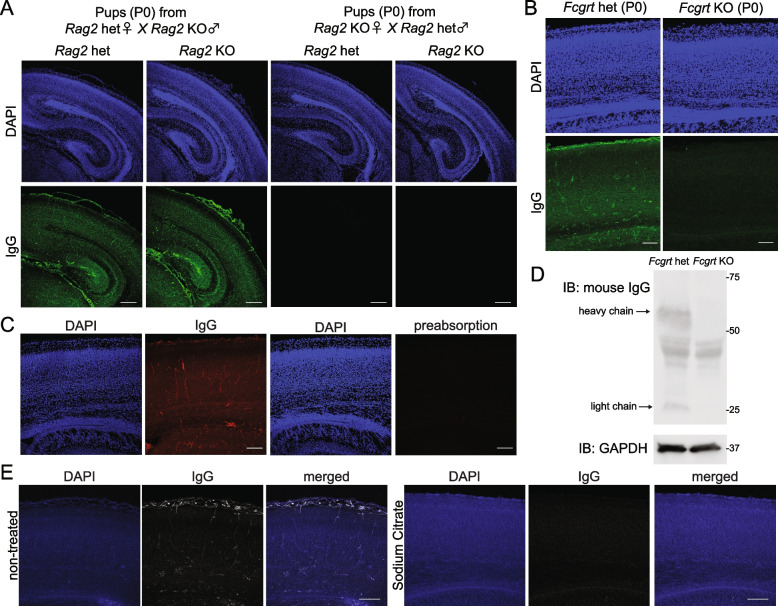
Maternal IgG is present in the developing mouse forebrain. **A**, **B** Immunohistochemistry of mouse IgG staining of forebrain samples from P0 pups of *Rag2* heterozygous (het) dams or *Rag2* KO dams (**A**) or P0 pups of cerebral cortex of *Fcgrt* het or *Fcgrt* KO mice (**B**). Scale bar: 200 μm (**A**), 100 μm (**B**). het: heterozygous, KO: knockout. (C) Preabsorption of anti-mouse IgG antibody with mouse IgG abolished the positive signal in the P0 ICR mouse forebrain. Scale bar: 100 μm. **D** The presence of IgG in the P0 forebrain of *Fcgrt* het or *Fcgrt* KO mice was assessed by Western blotting. **E** Immunohistochemistry of mouse IgG staining of a E18 cortical specimen with or without autoclaving using sodium citrate buffer. Scale bar: 100 μm

While it is known that the maternal IgG enters the embryonic brain, it is still unclear to what extent it is derived from the mother via the placenta or is produced by the embryos themselves. To address this issue, we used two types of Ig-deficient mice in this study: KO mice of the *Rag2* (recombination activating gene 2) gene and KO mice of the *Fcgrt* (Fc gamma receptor and transporter) gene, which encodes FcRn. The *Rag2* KO mice are deficient in Ig production, so when *Rag2* KO females are crossed with *Rag2* heterozygous males, the resulting *Rag2* KO pups would lack maternal IgG and are unable to produce IgG by themselves, while *Rag2* heterozygous pups lack maternal IgG but have the ability to produce IgG. In contrast, pups of *Rag2* heterozygous females and *Rag2* KO males would receive maternal IgG with the ability to produce IgG themselves if the pups’ genotype is heterozygous. As a result, immunohistochemical analyses clearly showed that postnatal day (P) 0 pups from *Rag2* KO dams completely lacked the IgG signal, regardless of the pups’ genotype (Fig. 1A), suggesting that IgG in the developing brain is basically derived from their mothers and not produced by the embryos themselves. The *Fcgrt* KO mice are defective in placental IgG transport because the FcRn protein is expressed at the embryonic syncytiotrophoblast and is actively used to transport maternal IgG to the embryonic blood. Similar to the *Rag2* KO model, no IgG signal was detected in the *Fcgrt* KO pups (Fig. 1B). This IgG staining observed in the brain of wild-type mice was completely abolished by preabsorption of anti-mouse IgG antibody with mouse IgG, so the signal appears to be specific for IgG (Fig. 1C). Western blot analysis also confirmed that *Fcgrt* KO brains lacked both IgG heavy chain and light chain (Fig. 1D). These results suggest that IgG in the developing brain is maternally derived and that IgG produced by the embryos themselves, if present, is barely detectable.

These endogenous IgG signals are often a problem in immunohistochemistry using mouse antibodies. We investigated methods to remove these signals and found that autoclaving the sections at 105 °C for 10 min in sodium citrate buffer (pH 6.0) was useful (Fig. 1E).

### Developmental changes in the amount of maternal IgG in the brain

**Fig. 2:**
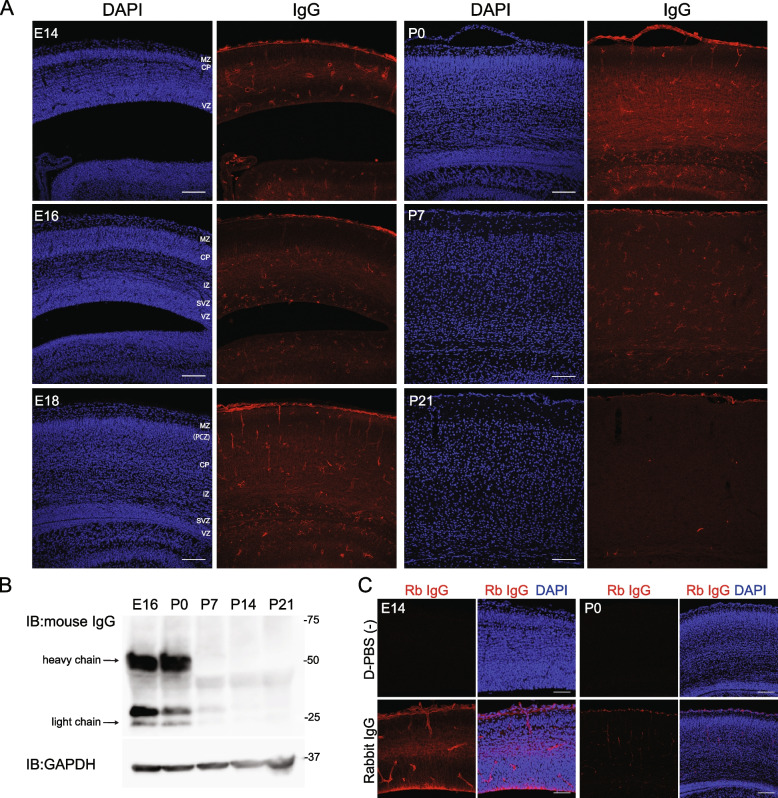
Distribution of maternal IgG in the developing mouse cerebral cortex. **A** Cerebral cortex from E14 to P21 was stained with anti-mouse IgG antibody. Scale bar: 100 μm. MZ: marginal zone, PCZ: primitive cortical zone, CP: cortical plate, IZ: intermediate zone, SVZ: subventricular zone, VZ: ventricular zone. **B** Representative Western blot images of IgG in the forebrain from E16 to P21. GAPDH was used as a loading control. **C** Assessment of BBB permeability by trans-cardiac perfusion of rabbit IgG into E14 or P0 ICR mice. Scale bar: 50 μm (E14), 100 μm (P0)

We next examined the developmental profile of maternal IgG levels in the brain. From E14 to P0, strong IgG signals were detected immunohistochemically in the cerebral cortex, which gradually decreased and almost disappeared by P21 (Fig. 2A). Western blot analysis using an anti-mouse IgG antibody revealed bands around the molecular weights of 50 k and 25 k, corresponding to the heavy and light chains of IgG, respectively (Fig. 2B). As expected, IgG levels were highest in the late embryonic stages and decreased dramatically after birth.

The BBB is known to be immature in embryonic stages and permeable to plasma proteins, allowing IgG entry into the embryonic brain up to E17.5, as demonstrated using infrared-labeled antibodies in C57BL/6 J, Balb/c, and NMRI (Naval Medical Research Institute) mice, although germ-free mice have a more permeable BBB [5]. To assess the permeability of IgG into the brain of ICR mice, we performed transcardiac perfusion with rabbit IgG in wild-type ICR mice and observed IgG penetration into the brain parenchyma at E14, but barely at P0 (Fig. 2C). The limited duration of maternal IgG presence in the brain can be attributed to the developmental time course of BBB maturation and the half-life of IgG (in the adult mice, 6–8 days for IgG1 and G3, 4–6 days for IgG2b) [14]. These findings suggest that maternal IgG is transferred to the developing brain almost exclusively during the embryonic period.

### Distribution of maternal IgG in the developing mouse cerebral cortex

**Fig. 3:**
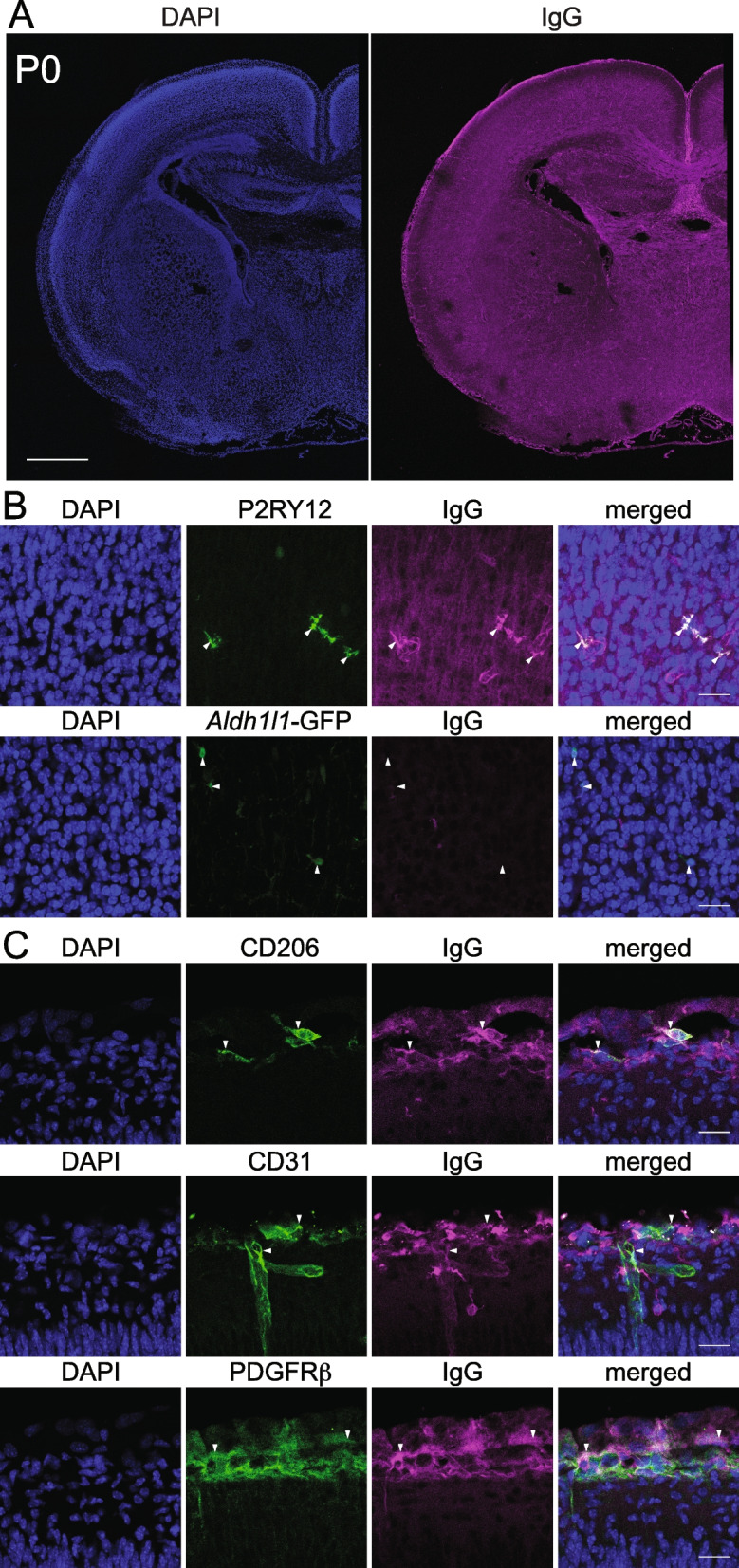
IgG positive cells in P0 forebrain. **A** Distribution of IgG in P0 ICR forebrain. Scale bar: 500 μm. **B** P2RY12-positive microglia were stained with anti-mouse IgG antibody, whereas *Aldh1l1*-GFP-positive astrocytes showed no IgG signal, as shown by immunohistochemistry of the cerebral cortex of P0 mice. Scale bar: 20 μm. **C** IgG signals on the meningeal surface were assessed by co-immunostaining of anti-mouse IgG antibody with anti-CD206, CD31, and PDGFRβ antibodies. Scale bar: 20 μm

We next examined the distribution of maternal IgG in the brain in more detail. Mouse brain samples stained with an anti-mouse IgG antibody at P0 showed IgG deposition throughout the forebrain, with strong signals observed along axon bundles such as the white matter of the cerebral cortex, corpus callosum (Fig. 3A), and internal capsule (data not shown).

At E14 and E16, the intermediate zone (IZ) was stained with an anti-IgG antibody, and at E18, the marginal zone (MZ) and cortical plate (CP) began to show IgG staining. Within the CP, IgG signal was weak in the primitive cortical zone (PCZ) [15, 16], where immature neurons are densely packed just beneath the MZ. Similarly, cell-dense regions such as the ventricular zone (VZ), hippocampal pyramidal cell layer, and granular cell layer were weak in the IgG signal at P0 (Figs. 1A, 2A, and 3A). At P7, the entire cerebral cortical wall exhibited ubiquitous but weak staining with an anti-IgG antibody (Fig. 2A).

### Microglia and meningeal cells are positive for IgG

Next, we examined the cell types that were strongly positive for IgG. Immunostaining for a microglial marker, P2RY12, showed that P2RY12 + cells were strongly stained for IgG at P0, suggesting that microglia take up IgG (Fig. 3B). In contrast, astrocytes were negative for IgG, as shown by staining of the aldehyde dehydrogenase 1 family member L1 (*Aldh1l1*)-GFP transgenic mice, in which astrocytes are positive for GFP (Fig. 3B). At P14, the IgG signal on P2RY12 + cells became weak, and completely disappeared at P21 (Additional file 2: Figure S2). With regard to the meningeal surface, where strong IgG signals were also observed, macrophages located at the brain surface, called BAMs, were immunoreactive for IgG, as shown by labeling for the mannose receptor, CD206. In addition, CD31 (platelet endothelial cell adhesion molecule 1, PECAM-1) + endothelial cells and PDGFRβ (platelet-derived growth factor receptor beta polypeptide β)  + cells, which may include fibroblasts, pericytes, and smooth muscle cells, were also stained with an anti-IgG antibody (Fig. 3C).

### Microglia and BAMs take up maternal IgG via the Fcγ receptor

**Fig. 4:**
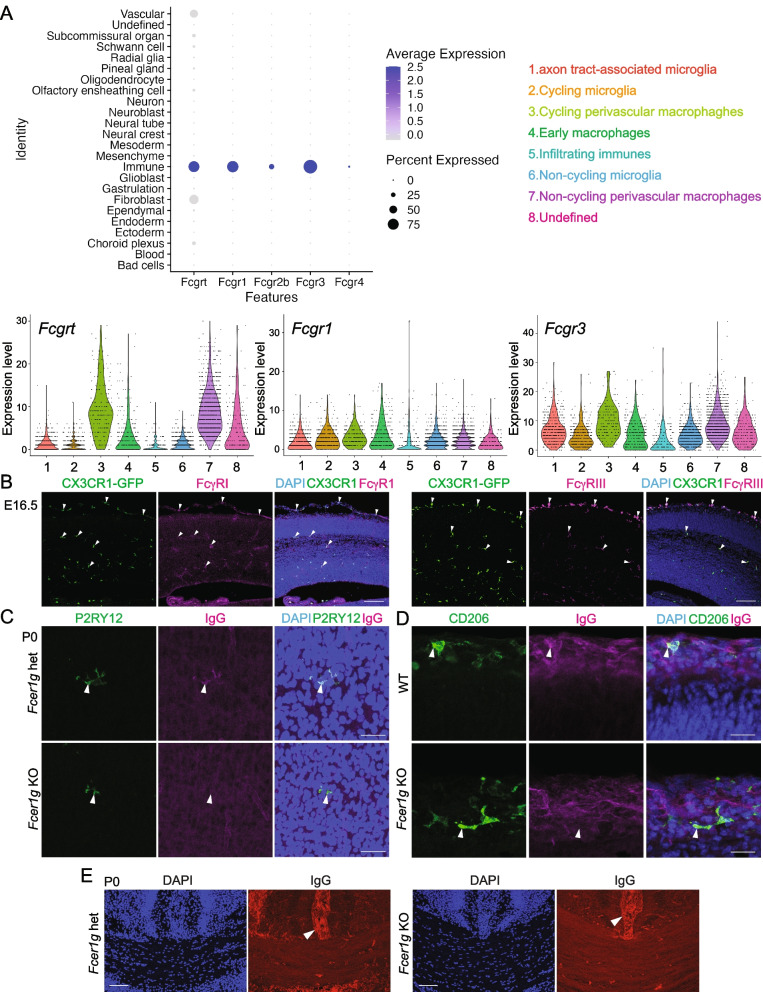
Fcγ receptor dependence for IgG uptake in the developing forebrain. **A** Fcγ receptor expression analysis using single-cell RNA-seq data from E7-E18.5 mouse brain. The “Immune” cluster is divided into 8 classes. The mRNA expression of *Fcgrt, Fcgr1,* and *Fcgr3* in different types of microglia and macrophages is shown in violin plots. **B** FcγRI and FcγRIII protein expression in CX3CR1-GFP mice at E16.5. Scale bar: 100 μm. **C**, **D** Co-immunohistochemistry of P0 cerebral cortex. IgG signals on P2RY12-positive microglia and CD206-positive BAMs are nearly abolished in *Fcer1g* KO mice. Scale bar: 25 μm. **E** Immunohistochemistry of P0 mice. IgG signals are observed in the meninges as indicated by the arrowhead and axon bundles at the corpus callosum in *Fcer1g* KO mice. Scale bar: 100 μm

Next, we investigated how brain cells take up IgG. Microglia and BAMs are known to take up maternal IgG in an Fcγ receptor-dependent manner in adulthood [17], but the expression of these Fc receptors in the developing brain has not been reported. By re-analyzing single-cell RNA-seq data from E7-E18.5 brains [10], we found that immune cells in the brain strongly express mRNAs for *Fcgrt*, *Fcgr1* (encoding FcγRI), *Fcgr2b* (encoding FcγRIIb), and *Fcgr3* (encoding FcγRIII), compared to vascular cells and fibroblasts. The immune cell cluster in the original data is subdivided into eight classes by several markers: (1) axon tract-associated microglia, (2) cycling microglia, (3) cycling perivascular macrophages, (4) early macrophages, (5) infiltrating immune cells, (6) non-cycling microglia, (7) non-cycling perivascular macrophages, and (8) undefined immune cells (Fig. 4A). While the expression level of *Fcgr1* is almost the same between microglia and macrophages, *Fcgrt* and *Fcgr3* are highly expressed in perivascular macrophages compared to microglia. Immunohistochemical analysis showed that microglia and BAMs in the developing cortex expressed FcγRI and FcγRIII proteins, and the signal of FcγRIII staining was stronger in BAMs compared to microglia (Fig. 4B). Both FcγRI and FcγRIII use the same intracellular subunit FcRγ, encoded by *Fcer1g*, to transduce activation signals, and the FcRγ chain is known to be essential for both surface expression and function of FcγRI and FcγRIII [18]. Therefore, we generated *Fcer1g* KO mice using i-GONAD (Additional file 1: Figure S1B). Immunohistochemical analysis of the P0 cerebral cortex of *Fcer1g* KO mice confirmed that FcγRI and FcγRIII proteins were undetectable (or barely detectable) in microglia (Additional file 3: Figure S3A, 3B). Notably, IgG signals were not or barely detected on the P2RY12 + microglia and CD206 + BAMs in the *Fcer1g* KO brain (Fig. 4C, D), although IgG signals were still observed clearly on axon bundles and meninges (Fig. 4E). These results suggest that microglia and BAMs capture IgG in an FcRγ-dependent manner and that the deposition of IgG on axon bundles and meninges is independent of the FcRγ.

### Cortical interneurons are decreased postnatally in maternal IgG-deficient models

**Fig. 5:**
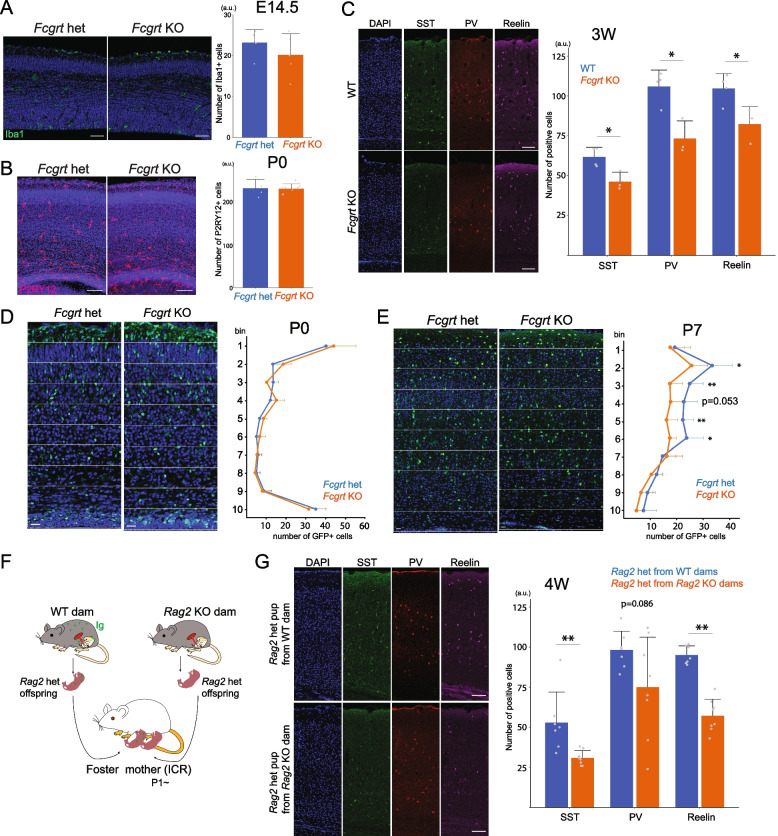
Phenotype of maternal IgG-deficient mice. **A** Iba1-positive cells in the cerebral cortex of *Fcgrt* heterozygous (het) and *Fcgrt* KO mice at E14.5. *Fcgrt* het: *n* = 5, *Fcgrt* KO: *n* = 5. Scale bar: 50 μm. **B** P2RY12-positive cells in the cerebral cortex of *Fcgrt* het and *Fcgrt* KO male mice at P0. *Fcgrt* het: *n* = 4, *Fcgrt* KO: *n* = 8. Scale bar: 100 μm. **C** SST-, PV-, and Reelin-positive cells in the somatosensory cortex of wild-type (WT) and *Fcgrt* KO mice at 3 weeks. WT: *n* = 4, *Fcgrt* KO: *n* = 3. Scale bar: 100 μm. **D**, **E** GAD-GFP positive cells in the somatosensory cortex of *Fcgrt* het and *Fcgrt* KO mice at P0 (*Fcgrt* het: *n* = 5, *Fcgrt* KO: *n* = 6) and P7 (*Fcgrt* het: *n* = 7, *Fcgrt* KO: *n* = 11). GAD-GFP mice were crossed with *Fcgrt* KO mice to visualize interneurons by GFP signal. Scale bar: 20 μm. **F** Experimental scheme for rearing *Rag2* het pups from wild-type or *Rag2* KO dams. **G** SST-, PV-, and Reelin-positive cells in the somatosensory cortex of *Rag2* het pups from wild-type dams or *Rag2* KO dams raised by wild-type ICR mice for 3 weeks. *Rag2* het pups from WT dams: *n* = 7, *Rag2* het pups from *Rag2* KO dams: *n* = 8. Scale bar: 100 μm. *P* values from Welch’s *t*-test are shown. *, *P* < 0.05; **, *P* < 0.01. a.u.: arbitrary unit

Finally, we investigated the influence of maternal IgG deficiency during brain development. Considering that microglia take up IgG via the FcγR, we first compared the number of cortical microglia at E14.5 and P0 between *Fcgrt* KO and heterozygous mice. As a result, we didn’t find any obvious differences (Fig. 5A, B). We then counted the number of different cell types in the cortex and found that SST + , PV + , and reelin + interneurons were all decreased in the somatosensory cortex of *Fcgrt* KO mice compared to heterozygous mice at 3 weeks postnatal age (Fig. 5C). Since the number of cortical interneurons at 3 weeks postnatal would be determined by the degree of neuronal production in the embryonic stages, migration into the cortex [19], and programmed cell death that occurs postnatally [20], we decided to examine the time point at which interneurons begin to decrease. Since several subtype markers such as PV are not expressed in interneurons at early stages, we crossed *Fcgrt* KO mice with GAD-GFP mice to visualize all interneurons. Quantitative analyses showed that cortical interneurons were decreased in *Fcgrt* KO mice at P7 but not at P0 (Fig. 5D, E), indicating that interneurons begin to decrease after birth, supporting the possibility that maternal IgG is necessary for the offspring cortical interneurons as their number is adjusted by programmed cell death, which occurs in the first two postnatal weeks.

Given that FcRn is critical for the maintenance of not only IgG but also albumin by regulating transcytosis and recycling, and is also known to act as an immune receptor on dendritic cells and macrophages to facilitate antigen presentation of peptides derived from IgG immune complexes [21], the interneuron abnormalities observed in the *Fcgrt* KO mice could be attributable to factors other than IgG. To address this possibility, we next used the *Rag2* model, in which neonatal pups were reared by a wild-type ICR mother from P1 to eliminate the potential postnatal influence of maternal factors associated with *Rag2* KO and wild-type mothers (Fig. 5F). Pups from *Rag2* KO mothers showed a reduction in SST + and reelin + interneurons at 4 weeks postnatal age (Fig. 5G). Although the reduction in PV + interneurons was not statistically significant (*p* = 0.086), there was a trend toward a reduction in pups from *Rag2* KO mothers. These results suggest that the maternal IgG is critical for the postnatal maintenance of cortical interneurons.

## Discussion

In this study, we show that IgG in the developing brain is basically maternal and not produced by the pups themselves. Although it is difficult to completely exclude the possibility that the embryonic and neonatal pups can produce an undetectable amount of IgG, it is likely that the pups do not produce enough IgG to be detectable by immunohistochemistry and Western blot analysis. The IgG is detected mainly on axon bundles, meningeal cells, and microglia. FcRγ, which is shared by FcγRI and FcγRIII, the Fcγ receptors expressed on microglia and BAMs, is required for these cells to take up maternal IgG, while FcRγ is not essential for the other IgG + cells, including axon bundles. These results suggest that maternal IgG may be used in multiple ways by different cell types in the developing brain. Although the presence of maternal IgG does not affect the number of microglia, it is important to maintain an adequate number of cortical interneurons after birth.

Positive immunohistochemical signals with an anti-IgG antibody on developing brains have been reported for the early-generated neurons, especially cortical subplate neurons, during developmental stages in cats, rats, and mice [22–26]. IgG immunoreactivity in the developing brain has been interpreted as the presence of Ig-like molecules produced by neurons. Although much effort was made to detect the mRNA in neurons, it was not successful, leading to the idea that subplate neurons might selectively take up Igs from the serum. Regarding the origin of IgG in the embryonic brain, a previous study reported that brain samples from recombination activating gene 1 (*Rag1*) heterozygous embryos carried by *Rag1* KO females did not contain IgG, as shown by Western blot analysis, suggesting a maternal origin of the IgG [26]. However, since *Rag1* KO mice are immunodeficient, lacking B cells, T cells, and NKT cells, the possibility remained that some developmental abnormalities caused by the immunodeficiency might have secondarily led to the absence of IgG signals in the brain. Therefore, in this study, we examined two mouse models to clarify the origin of the embryonic brain IgG: an IgG production-deficient mouse (*Rag2* KO mice) and an IgG transport-deficient mouse (*Fcgrt* KO mice). As a result, we clearly showed that embryonic brain IgG was not detectable when the embryos could not receive the maternal IgG.

Previous studies indicated that the peak of IgG in the brain was around E16, as shown by comparing the signal intensity of brain samples from E12 to P1 using Western blot [26]. In our study, we compared IgG levels from the embryonic stage to adolescence and found abundant IgG in the brain during the embryonic and neonatal periods, with levels gradually decreasing postnatally and almost disappearing by P21. The comparison of IgG levels between E16 and P0 is challenging because of the strong correlation between fetal serum IgG levels and maternal serum IgG levels, which show a wide range of variation [1]. Although the signal intensity observed in the Western blot almost disappeared by P21, more sensitive ELISA assays were able to detect low levels of IgG still present in the adult brain (data not shown).

The presence of maternal IgG poses a challenge to immunohistochemistry when using primary antibodies derived from mice as hosts, particularly when studying signals in the meninges and microglia. Compared to the preparation of primary antibodies directly conjugated to a fluorescent dye, our autoclaving method is time and cost-saving and can be widely used to study the developing mouse brain using immunohistochemical techniques.

We observed a robust IgG signal on meninges, microglia, and axon bundles. Notably, the signals on microglia and BAMs were absent or barely detectable in *Fcer1g* KO mice, whereas the signals at other sites, including axon bundles, remained intact. The signals on axon bundles were never observed in the IgG-deficient model, and staining was specifically eliminated by preabsorption of the IgG antibodies with purified mouse IgG, suggesting that IgG is captured by an unknown receptor on axon bundles. Further analysis is required to identify the molecule(s) essential for IgG deposition in the developing brain.

The primary function of IgG is to protect the host from invading pathogens such as bacteria. It is well established that the maternal IgG is transferred to the embryo/fetus to provide protection against pathogens until the embryo/fetus has developed to produce sufficient antibodies. However, the absence of obvious infection and inflammation in the healthy developing brain raises questions about the role of IgG, despite its risk, in the physiological state. Evidence suggests that breast milk given to very low birth weight infants partially rescues the decline in IQ [27], suggesting that some factors, including IgG, in milk may reach the CNS across the immature BBB and support proper brain development. In addition, monomeric IgG has been shown to have neuroprotective effects by promoting microglial endocytosis and tumor necrotic factor-α (TNF-α) production at physiological levels (i.e., 0.2–20 μg/ml) in adult mice [28]. Therefore, maternally derived IgG in the embryonic brain may have an unknown function in supporting neurons during brain development, in addition to its protective role against infection. In this study, we report that maternal IgG is necessary to maintain an adequate number of interneurons in the postnatal cortex. The number of cortical interneurons is determined by several developmental events. The first is when the interneurons are generated during the mid to late embryonic stages within the ganglionic eminences and the preoptic area [19, 29]. They then migrate tangentially to the neocortex through both the superficial migratory stream (SMS) and the deep migratory stream (DMS) [30–32]. The former, located just beneath the meningeal surface, shows strong IgG deposition. Conversely, the latter pathway passes through the subplate and intermediate zone, where IgG signal is observed on axon bundles. Therefore, we hypothesized that maternal IgG deficiency might impair the migration of interneurons to the cortex. However, our results indicate that the number of interneurons remains normal at P0, suggesting that neuronal production and migration are not impaired in the maternal IgG-deficient models. Third, interneurons are known to undergo programmed cell death during a critical window of postnatal development, decreasing by nearly 30% between P5 and P10 and remaining nearly constant into adulthood [33]. Since we observed that the number of cortical interneurons was reduced postnatally (P7) in the *Fcgrt* KO mice, we assume that maternal IgG is important in this adjustment process of interneuron number through programmed cell death.

The mechanism by which maternal IgG affects the interneurons is still unknown since re-analysis of the single-cell RNA-seq data suggests that cortical interneurons themselves do not express conventional Fcγ receptors (data not shown). The maternal IgG may indirectly affect postnatal interneuron survival via other cell types. Candidate cells are microglia and BAMs, which are strongly stained by IgG antibodies. Indeed, microglia have the ability to produce and release a wide range of factors, spanning from cytotoxic mediators such as cytokines, proteases, free radicals, and glutamate agonists, to trophic factors such as nerve growth factor, IL-10, and insulin-like growth factor 1 (IGF1) [34]. These factors have the potential to exert both deleterious and beneficial effects on the surrounding neurons [34]. Microglial depletion by pexidartinib (PLX) injection from P1-P15 does not change the number of PV + cells, although it causes altered inhibitory and excitatory synaptic connectivity [35], and microglia regulate the density of SST + interneuron synapses postnatally [36]. Another study also reported that microglial depletion by intraperitoneal injection of anti-CSF-1R (colony-stimulating factor 1 receptor) Ab in E6.5 and E7.5 dams resulted in slight changes in the laminar positioning of PV + interneurons at P20 [37]. Moreover, intravenous Ig induces a neuroprotective phenotype in microglia by preventing neuronal cell death in ischemic stroke [38]. These data suggest that microglia may play a role in regulating the number of interneurons, although the precise mechanisms remain unclear. Our preliminary microarray analysis of microglia from a maternal IgG-deficient model did not reveal significant changes in activation status and phagocytic activity. In addition, flow cytometry analysis showed that the expression levels of CD11b, an activation marker, in *Fcgrt* heterozygous and *Fcgrt* KO microglia, were almost similar between genotypes. One of the critical experiments is to evaluate the number of interneurons in *Fcer1g* KO mice to determine the dependence on FcRγ. If *Fcer1g* KO mice have normal numbers of interneurons, it is thought that maternal IgG affects the interneurons independently of microglia and BAMs. In this case, the maternal IgG on other structures, including axon bundles and meningeal membranes, as well as some peripheral effects of maternal IgG should be considered. Another candidate mechanism that could explain the reduction in interneuron numbers in IgG-deficient mice is mediated by cortical excitatory neurons because interneuron survival is known to depend on the activity of excitatory neurons during a critical window of postnatal development when excitatory synaptic input to individual interneurons predicts their survival or death [33].

While the overall survival and growth of our maternal IgG-deficient mice were nearly normal, it is important to evaluate behavioral changes in these mice, because alterations in interneurons have been implicated in neurodevelopmental disorders such as ASD [39] and Tourette syndrome [40]. Our data from open field tests using *Rag2* heterozygous male mice from *Rag2* heterozygous dams or *Rag2* KO dams showed that the time spent in the center was significantly longer in the latter group, although the total distance traveled was comparable between the two groups (data not shown). However, it is important to note that these results may be influenced by defects in cytokines produced by maternal B cells and T cells in the *Rag2* KO mice, as well as potential effects caused by differences in postnatal rearing conditions. Further analysis using the fostered *Rag2* model and the *Fcgrt* model is needed to more precisely delineate the effects on behavior.

In terms of clinical implications, there are no reports linking maternal agammaglobulinemia to neurodevelopmental disorders in their offspring. Given that Ig replacement therapy is commonly administered to patients with agammaglobulinemia, it is plausible that the treatment received by the mother may have mitigated the effects of deficient maternal IgG on the child’s neurodevelopment [41]. It would be important to determine the appropriate concentration of maternally derived IgG to maintain the proper number of interneurons. In addition, there are a limited number of papers that specifically address the maternal genotype to assess the offspring phenotype. For example, in the reported phenotype of social recognition memory and impaired emotional behavior in *Rag1* KO mice may have used a mixture of pups from KO and heterozygous mothers [42], potentially confounding the interpretation of the results. Therefore, it is important to pay close attention to the evaluation of maternal influence on the offspring.

## Conclusions

Here, we demonstrate that maternal IgG distributed in the developing brain is preferentially taken up by microglia and BAMs in an FcRγ-dependent manner, and by axon bundles and meningeal cells in an FcRγ-independent manner. Maternal IgG plays a critical role in the postnatal maintenance of cortical interneurons.

### Supplementary Information

Additional file 1: Figure S1. Genome editing of Fcgrt and Fcer1g using i-GONAD. (A, B) Strategy of complete deletion of the coding region of Fcgrt (encoding FcRn) (A) and Fcer1g (encoding FcRγ) (B). Fcgrt contains 7 exons and the start codon is located in exon 2. Fcgrt KO mice lack the entire coding region. Fcer1g contains 5 exons and Fcer1g KO mice completely lack the coding region.

Additional file 2: Figure S2. Co-immunostaining of P2RY12 and IgG in the cerebral cortex of P14 and P21 wild-type ICR mice. At P14, P2RY12-positive microglia are weakly positive for IgG, whereas the IgG signal disappeared at P21. Scale bar: 25 µm.

Additional file 3: Figure S3. FcRγ protein expression in microglia in the cerebral cortex of Fcer1g het and Fcer1g KO mice at P0. (A, B) In Fcer1g KO mice, the protein expression of FcγRI (A) and FcγRIII (B) disappears in P2RY12-positive microglia. Scale bar: 25 µm.

Additional file 4: Figure S4. Original full-length gel and blot images in Fig. 1D. Red dashed boxes indicate the sections of the gels and blot in Fig. 1D.

Additional file 5: Figure S5. Original full-length gel and blot images in Fig. 2B. Red dashed boxes indicate the sections of the gels and blot in Fig. 2B.

## Data Availability

All data necessary to evaluate the conclusions of the paper are included in the paper.

## Abbreviations

ADHD: Attention-deficit/hyperactivity disorder
Aldh1l1: Aldehyde dehydrogenase 1 family member L1
ASD: Autism spectrum disorder
BAM: Border-associated macrophage
BBB: Blood-brain barrier
CASPR2: Contactin-associated protein 2
CP: Cortical plate
CRMP1: Collapsin response mediator protein 1
CSF-1R: Colony-stimulating factor 1 receptor
DAPI: 4,6-Diamidino-2-phenylindole, dihydrochloride
E: Embryonic day
FcRγ: Fc receptor γ chain
FcRn: Neonatal Fc receptor
i-GONAD: Improved genome-editing via oviductal nucleic acid delivery
Ig: Immunoglobulin
IGF1: Insulin-like growth factor 1
IgG: Immunoglobulin G
IZ: Intermediate zone
KI: Knock-in
KO: Knock-out
MZ: Marzinal zone
P: Postnatal day
PBS: Phosphate buffered saline
PBST: PBS containing 0.05% Triton X-100
PCZ: Primitive cortical zone
PDGFRβ: Platelet-derived growth factor receptor beta
PFA: Paraformaldehyde
PLX: Pexidartinib
PV: Parvalbumin
PVDF: Polyvinyldifluoride
P2RY12: Purinergic receptor P2Y, G-protein coupled 12
Rag1: Recombination activating gene 1
Rag2: Recombination activating gene 2
RT: Room temperature
SDS-PAGE: Sodium dodecyl sulfate–polyacrylamide gel electrophoresis
SST: Somatostatin
STIP1: Stress-induced phosphoprotein 1
TNF-α: Tumor necrotic factor-α
VZ: Ventricular zone
YBX1: Y-box binding protein 1

## References

[1] Malek A, Sager R, Kuhn P, Nicolaides KH, Schneider H (1996). Evolution of maternofetal transport of immunoglobulins during human pregnancy. Am J Reprod Immunol.

[2] Mishra A, Lai GC, Yao LJ, Aung TT, Shental N, Rotter-Maskowitz A (2021). Microbial exposure during early human development primes fetal immune cells. Cell.

[3] Han VX, Patel S, Jones HF, Dale RC (2021). Maternal immune activation and neuroinflammation in human neurodevelopmental disorders. Nat Rev Neurol.

[4] Bagnall-Moreau C, Spielman B, Brimberg L (2023). Maternal brain reactive antibodies profile in autism spectrum disorder: an update. Transl Psychiatry.

[5] Braniste V, Al-Asmakh M, Kowal C, Anuar F, Abbaspour A, Toth M (2014). The gut microbiota influences blood-brain barrier permeability in mice. Sci Transl Med..

[6] Morimoto K, Nakajima K (2019). Role of the immune system in the development of the central nervous system. Front Neurosci.

[7] Cahoy JD, Emery B, Kaushal A, Foo LC, Zamanian JL, Christopherson KS (2008). A transcriptome database for astrocytes, neurons, and oligodendrocytes: a new resource for understanding brain development and function. J Neurosci.

[8] Tamamaki N, Yanagawa Y, Tomioka R, Miyazaki J, Obata K, Kaneko T (2003). Green fluorescent protein expression and colocalization with calretinin, parvalbumin, and somatostatin in the GAD67-GFP knock-in mouse. J Comp Neurol.

[9] Gurumurthy CB, Sato M, Nakamura A, Inui M, Kawano N, Islam MA (2019). Creation of CRISPR-based germline-genome-engineered mice without ex vivo handling of zygotes by i-GONAD. Nat Protoc.

[10] La Manno G, Siletti K, Furlan A, Gyllborg D, Vinsland E, Mossi Albiach A (2021). Molecular architecture of the developing mouse brain. Nature.

[11] Hao Y, Hao S, Andersen-Nissen E, Mauck WM, Zheng S, Butler A (2021). Integrated analysis of multimodal single-cell data. Cell..

[12] Schindelin J, Arganda-Carreras I, Frise E, Kaynig V, Longair M, Pietzsch T (2012). Fiji: an open-source platform for biological-image analysis. Nat Methods.

[13] Haase R, Royer LA, Steinbach P, Schmidt D, Dibrov A, Schmidt U (2020). CLIJ: GPU-accelerated image processing for everyone. Nat Methods.

[14] Vieira P, Rajewsky K (1988). The half-lives of serum immunoglobulins in adult mice. Eur J Immunol..

[15] Sekine K, Honda T, Kawauchi T, Kubo K, Nakajima K (2011). The outermost region of the developing cortical plate is crucial for both the switch of the radial migration mode and the Dab1-dependent "inside-out" lamination in the neocortex. J Neurosci.

[16] Sekine K, Kawauchi T, Kubo K, Honda T, Herz J, Hattori M (2012). Reelin controls neuronal positioning by promoting cell-matrix adhesion via inside-out activation of integrin alpha5beta1. Neuron.

[17] Hazama GI, Yasuhara O, Morita H, Aimi Y, Tooyama I, Kimura H (2005). Mouse brain IgG-like immunoreactivity: strain-specific occurrence in microglia and biochemical identification of IgG. J Comp Neurol.

[18] van Vugt MJ, Heijnen IA, Capel PJ, Park SY, Ra C, Saito T (1996). FcR gamma-chain is essential for both surface expression and function of human Fc gamma RI (CD64) in vivo. Blood.

[19] Nakajima K (2007). Control of tangential/non-radial migration of neurons in the developing cerebral cortex. Neurochem Int.

[20] Southwell DG, Paredes MF, Galvao RP, Jones DL, Froemke RC, Sebe JY (2012). Intrinsically determined cell death of developing cortical interneurons. Nature.

[21] Pyzik M, Sand KMK, Hubbard JJ, Andersen JT, Sandlie I, Blumberg RS (2019). The neonatal fc receptor (FcRn): a misnomer?. Front Immunol.

[22] Fairen A, Smith-Fernandez A, Marti E, DeDiego I, de la Rosa EJ (1992). A transient immunoglobulin-like reactivity in the developing cerebral cortex of rodents. NeuroReport.

[23] Henschel R, Wahle P (1994). The SP1 antigen in subplate neurons of the developing cat cortex is an immunoglobulin-like molecule. Eur J Neurosci.

[24] Dunn JA, Kirsch JD, Naegele JR (1995). Transient immunoglobulin-like molecules are present in the subplate zone and cerebral cortex during postnatal development. Cereb Cortex.

[25] Upender MB, Dunn JA, Wilson SM, Naegele JR (1997). Immunoglobulin molecules are present in early-generated neuronal populations in the rat cerebral cortex and retina. J Comp Neurol.

[26] Weiner JA, Chun J (1997). Maternally derived immunoglobulin light chain is present in the fetal mammalian CNS. J Neurosci.

[27] Vohr BR, Poindexter BB, Dusick AM, McKinley LT, Wright LL, Langer JC (2006). Beneficial effects of breast milk in the neonatal intensive care unit on the developmental outcome of extremely low birth weight infants at 18 months of age. Pediatrics.

[28] Hulse RE, Swenson WG, Kunkler PE, White DM, Kraig RP (2008). Monomeric IgG is neuroprotective via enhancing microglial recycling endocytosis and TNF-alpha. J Neurosci.

[29] Kanatani S, Honda T, Aramaki M, Hayashi K, Kubo K, Ishida M (2015). The COUP-TFII/Neuropilin-2 is a molecular switch steering diencephalon-derived GABAergic neurons in the developing mouse brain. Proc Natl Acad Sci U S A.

[30] Tanaka DH, Nakajima K (2012). Migratory pathways of GABAergic interneurons when they enter the neocortex. Eur J Neurosci.

[31] Marin O, Rubenstein JL (2001). A long, remarkable journey: tangential migration in the telencephalon. Nat Rev Neurosci.

[32] Morimoto K, Tabata H, Takahashi R, Nakajima K. Interactions between neural cells and blood vessels in central nervous system development. Bioessays. 2023:e2300091. 10.1002/bies.202300091. 10.1002/bies.202300091 38135890

[33] Wong FK, Bercsenyi K, Sreenivasan V, Portales A, Fernandez-Otero M, Marin O (2018). Pyramidal cell regulation of interneuron survival sculpts cortical networks. Nature.

[34] Marin-Teva JL, Cuadros MA, Martin-Oliva D, Navascues J (2011). Microglia and neuronal cell death. Neuron Glia Biol.

[35] Favuzzi E, Huang S, Saldi GA, Binan L, Ibrahim LA, Fernandez-Otero M (2021). GABA-receptive microglia selectively sculpt developing inhibitory circuits. Cell..

[36] Gesuita L, Cavaccini A, Argunsah AO, Favuzzi E, Ibrahim LA, Stachniak TJ (2022). Microglia contribute to the postnatal development of cortical somatostatin-positive inhibitory cells and to whisker-evoked cortical activity. Cell Rep.

[37] Squarzoni P, Oller G, Hoeffel G, Pont-Lezica L, Rostaing P, Low D (2014). Microglia modulate wiring of the embryonic forebrain. Cell Rep.

[38] Haussler V, Daehn T, Rissiek B, Roth V, Gerloff C, Arumugam TV (2020). Intravenous immunoglobulin (IVIg) induce a protective phenotype in microglia preventing neuronal cell death in ischaemic stroke. Neuromolecular Med.

[39] Contractor A, Ethell IM, Portera-Cailliau C (2021). Cortical interneurons in autism. Nat Neurosci.

[40] Rapanelli M, Frick LR, Pittenger C (2017). The role of interneurons in autism and Tourette syndrome. Trends Neurosci.

[41] Perez EE, Orange JS, Bonilla F, Chinen J, Chinn IK, Dorsey M (2017). Update on the use of immunoglobulin in human disease: a review of evidence. J Allergy Clin Immunol.

[42] Rattazzi L, Piras G, Ono M, Deacon R, Pariante CM, D'Acquisto F (2013). CD4(+) but not CD8(+) T cells revert the impaired emotional behavior of immunocompromised RAG-1-deficient mice. Transl Psychiatry.

