# Supplementary material for: Maternal immunoglobulins are distributed in the offspring’s brain to support the maintenance of cortical interneurons in the postnatal period
Source: Inflamm Regen. 2024 May 15;44:24. doi: 10.1186/s41232-024-00336-3 (PMC11094934; doi:10.1186/s41232-024-00336-3)
Supplement: Supplementary file 1 — Additional file 1: Figure S1. Genome editing of Fcgrt and Fcer1g using i-GONAD. (A, B) Strategy of complete deletion of the coding region of Fcgrt (encoding FcRn) (A) and Fcer1g (encoding FcRγ) (B). Fcgrt contains 7 exons and the start codon is located in exon 2. Fcgrt KO mice lack the entire coding region. Fcer1g contains 5 exons and Fcer1g KO mice completely lack the coding region. [file 41232_2024_336_MOESM1_ESM.pdf]

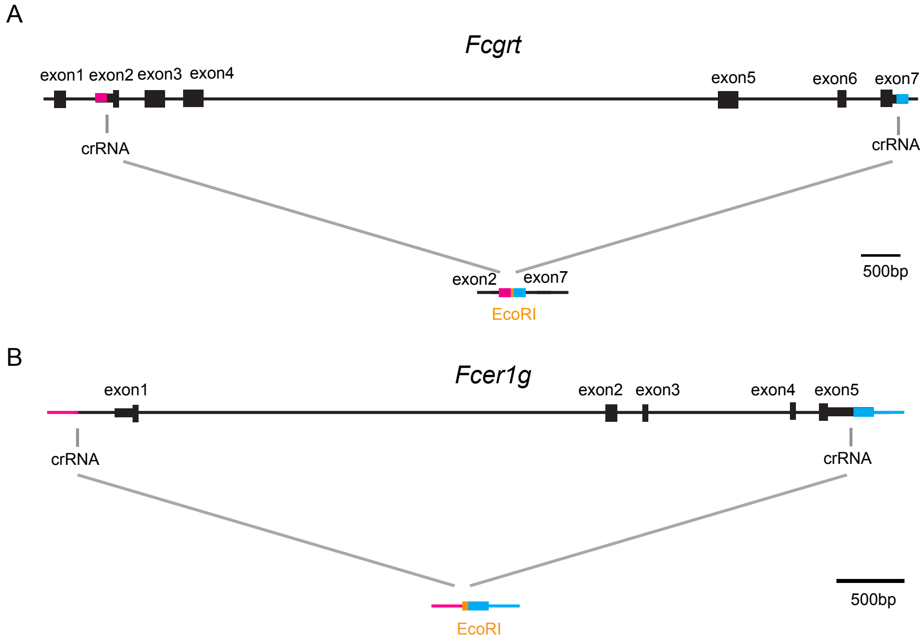

Fig. S1

Genome editing of *Fcgrt* and *Fcer1g* using i-GONAD. (A, B) Strategy of complete deletion of the coding region of *Fcgrt* (encoding FcRn) (A) and *Fcer1g* (encoding FcRγ) (B). *Fcgrt* contains 7 exons and the start codon is located in exon 2. *Fcgrt* KO mice lack the entire coding region. *Fcer1g* contains 5 exons and *Fcer1g* KO mice completely lack the coding region.
