# Supplementary figures and images for: Maternal immunoglobulins are distributed in the offspring’s brain to support the maintenance of cortical interneurons in the postnatal period
Source: Inflamm Regen. 2024 May 15;44:24. doi: 10.1186/s41232-024-00336-3 (PMC11094934; doi:10.1186/s41232-024-00336-3)

Fig.S2

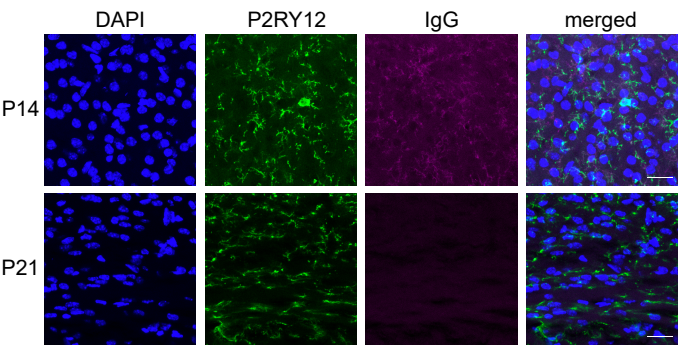

Supplement: Supplementary file 2 — Additional file 2: Figure S2. Co-immunostaining of P2RY12 and IgG in the cerebral cortex of P14 and P21 wild-type ICR mice. At P14, P2RY12-positive microglia are weakly positive for IgG, whereas the IgG signal disappeared at P21. Scale bar: 25 µm. [file 41232_2024_336_MOESM2_ESM.pdf]

Fig.S3

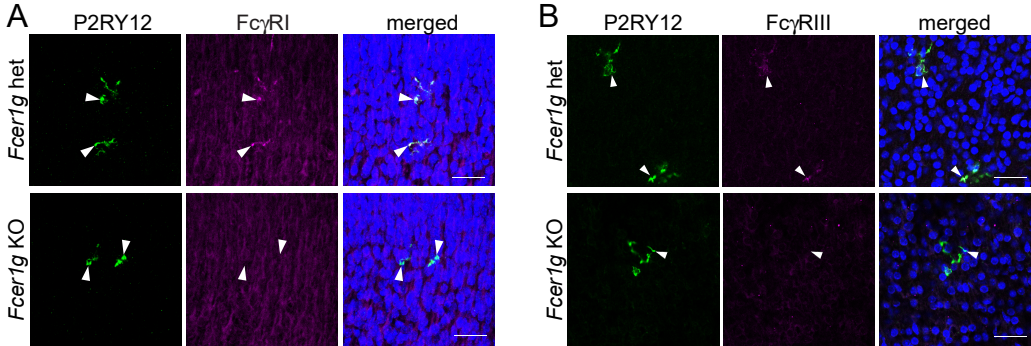

Supplement: Supplementary file 3 — Additional file 3: Figure S3. FcRγ protein expression in microglia in the cerebral cortex of Fcer1g het and Fcer1g KO mice at P0. (A, B) In Fcer1g KO mice, the protein expression of FcγRI (A) and FcγRIII (B) disappears in P2RY12-positive microglia. Scale bar: 25 µm. [file 41232_2024_336_MOESM3_ESM.pdf]
