# Supplementary material for: Maternal immunoglobulins are distributed in the offspring’s brain to support the maintenance of cortical interneurons in the postnatal period
Source: Inflamm Regen. 2024 May 15;44:24. doi: 10.1186/s41232-024-00336-3 (PMC11094934; doi:10.1186/s41232-024-00336-3)
Supplement: Supplementary file 4 — Additional file 4: Figure S4. Original full-length gel and blot images in Fig. 1D. Red dashed boxes indicate the sections of the gels and blot in Fig. 1D. [file 41232_2024_336_MOESM4_ESM.pdf]

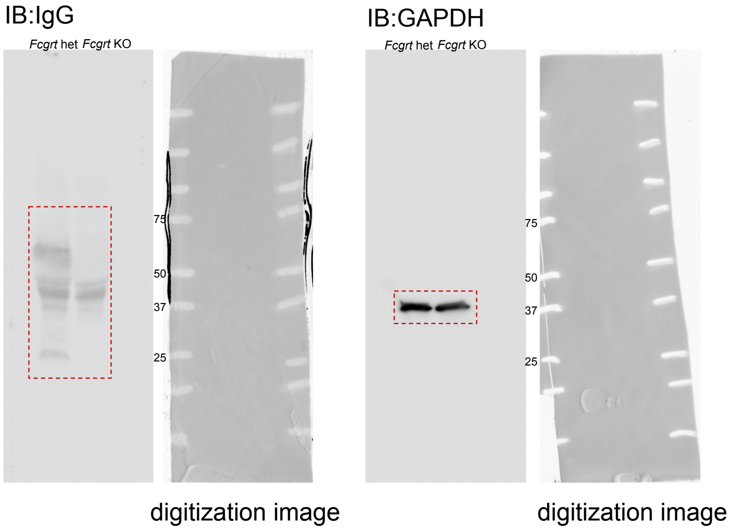

Fig. S4

Original full-length gel and blot images in Figure 1D. Red dashed boxes indicate the sections of the gels and blot in Figure 1D.
