# Supplementary material for: Maternal immunoglobulins are distributed in the offspring’s brain to support the maintenance of cortical interneurons in the postnatal period
Source: Inflamm Regen. 2024 May 15;44:24. doi: 10.1186/s41232-024-00336-3 (PMC11094934; doi:10.1186/s41232-024-00336-3)
Supplement: Supplementary file 5 — Additional file 5: Figure S5. Original full-length gel and blot images in Fig. 2B. Red dashed boxes indicate the sections of the gels and blot in Fig. 2B. [file 41232_2024_336_MOESM5_ESM.pdf]

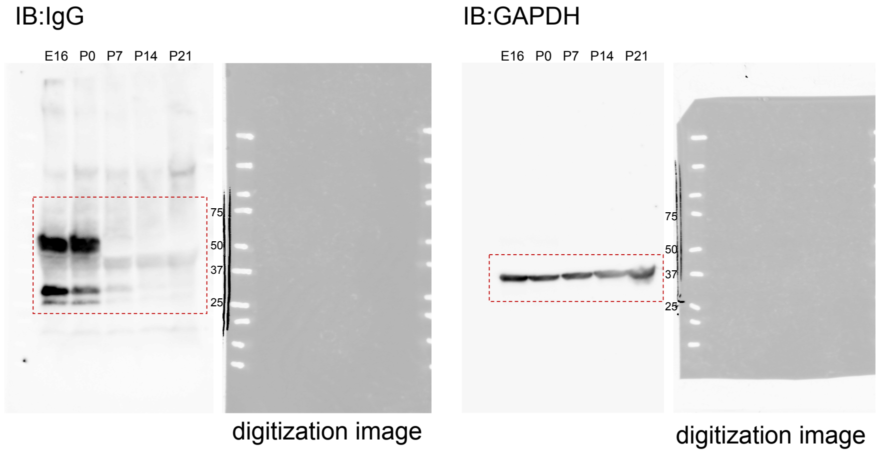

Fig. S5

Original full-length gel and blot images in Figure 2B. Red dashed boxes indicate the sections of the gels and blot in Figure 2B.
